# Radiological Control After Reduction Mammoplasty: Do Ultrasonographic Postoperative Changes Return to Normal in the Late Period?

**DOI:** 10.5152/eurasianjmed.2026.251260

**Published:** 2026-04-15

**Authors:** Nihal İşleyen

**Affiliations:** 1 Department of Plastic Aesthetic and Reconstructive Surgery, Atatürk University Faculty of Medicine, Erzurum, Türkiye

**Keywords:** Breast cancer, reduction mammoplasty, USG

## Abstract

**Background:**

Postoperative ultrasonographic follow-ups of 65 breast reduction cases performed from 2012 to 2020 were evaluated. It was discussed whether the postoperative changes in the late period were permanent and how permanent cases affected breast cancer screening.

**Methods:**

Sixty-five breast reduction cases performed in this clinic between 2012 and 2020 with at least 2 years of follow-ups were included in the study. Ultrasonography (USG) was administered to the patients before the operation and in the postoperative first month, sixth month, the first year, and every following year. While the patients under the age of 40 years were checked with USG only, mammography was also applied to the patients over the age of 40 together with USG during their controls.

**Results:**

Postoperative secondary changes observed in the USG findings returned to normal in 84.7% of the patients, while they were permanent in 15.3% of patients.

**Conclusion:**

Ultrasonography is one of the most reliable methods in postoperative breast evaluation and breast cancer screening.

**Main Points**

- Reduction mammoplasty is one of the most frequently used precedures today in plastic surgery practice.
- There may be difficulties in breast cancer screening after reduction mammoplasty surgery performed on young people.
- Postoperative changes in the breast may cause problems in screening.
- It is important to determine the time for postoperative changes to return to normal.
- Ultrasonography can also be used safely after breast reduction surgery.

## Introduction

Reduction mammoplasty (RM) is one of the most frequently used precedures today in plastic surgery practice. These operations are very effective and patient satisfaction is very high. For these reasons, the number of its applications increases day by day, and it is also applied to the younger population. Breast cancer (BC), which is in the third place among the most common cancers, is the most common malignant cancer in women, and one of every 8 women is diagnosed with BC during their lifetime.^1^ There may be some problems in screening patients after RM operations. In the present study, the ultrasonographic findings of the patients before and after the operation were compared. At the first step of the screening, it was determined whether the postoperative changes during the late period were permanent. Better recognition of postoperative radiological changes helps to make better differential diagnosis, and thus unnecessary biopsies can be avoided.

Breast cancer is a heterogeneous disease, and its pathogenesis remains unclear in most cases.^2^ Although the association of BRCA 1 and BRCA 2 gene mutations with BC has been shown, these gene mutations cannot be detected in every patient with BC.^3^

High survival and good prognosis are provided with early diagnosis and appropriate treatment.^2^ There is no consensus on the methods to be used for early diagnosis, and the age at which early diagnostic tests will begin. The general approach is to start screening tests at around the age of 35-40 with noninvasive methods. Ultrasound (USG), mammography (MG), magnetic resonance imaging (MRI), and positron emission tomography–computed tomography (PET-CT) are noninvasive techniques, while fine-needle aspiration biopsy, incisional and excisional biopsies are invasive methods used in the diagnosis of BC.^4^

Many authors recommend performing early diagnosis check-ups every 2 years after the age of 40 by USG alone or USG in conjunction with mammography.^5^ It is reported that USG is particularly useful in young patients and dense breasts.^5^ Ultrasound can assess the morphology, orientation, internal structure, and margins of lesions from multiple planes with high resolution in both predominantly fatty breasts and dense, glandular structures.^4^ Ultrasound is also economical and patient-friendly, does not emit radiation, and can be used safely in pregnant women. It is safe in distinguishing cystic, solid lesions and can be helpful in invasive procedures such as aspiration. Its disadvantages are that lesions and calcifications smaller than 1 cm are difficult to distinguish, and its being an operator-dependent method. Ultrasound works well only with a well-trained user.^5^

Although it is recommended to use USG together with mammography, not alone, some studies show that USG is superior to mammography in diagnosis.^6^

The characteristic USG images of malignant and benign lesions are summarized in Table 1.^7^^8^

The most common USG findings after breast operations are increased skin and subcutaneous thickness, seroma, hematoma, and fat necrosis. These findings are classified as changes secondary to the operation (CSO). Ultrasound is also important in determining early complications that need to be intervened. Cystic and solid pathologies may also be encountered in the postoperative USG imaging. Mammography with USG is routinely applied to patients over the age of 40. On the other hand, routine control of younger patients is made with USG, and mammography is used when necessary. In the postoperative period, when malignancy findings are suspected on USG, MRI is performed and early diagnosis should be made with excisional or incisional biopsy.

## Material and Methods

Ethical committee approval was obtained at the start of the study from Erzurum Regional Training and Research Hospital (Date: August 17, 2020; Number: 37732058-514.10). All patients were informed about the study and their informed consent was obtained.

A total of 65 breast reduction cases performed in this clinic between 2012 and 2020 with at least 2 years of follow-ups were included in the study. Patients who did not come to regular postoperative controls were excluded from the study. Ultrasound was administered to the patients before the operation decision was made. In the preoperative period, patients with suspected malignancy were first biopsied and breast reduction surgery was planned after malignancy was ruled out. Determining the location of benign lesions with USG is also effective in choosing the technique to be used and the pedicle for breast reduction. Ultrasound was performed to evaluate early complications in the postoperative 1 month and to evaluate seroma, hematoma, and infectious processes such as abscess that needed intervention. The patients were called for regular follow-up visits in the sixth month, the first year, and once in the following years. While the patients under the age of 40 were checked with USG only, MG was also applied to the patients over the age of 40 years together with USG during their controls. In case of suspected malignancy, mammography and MRI evaluations were performed. Early biopsy was recommended to the patients in the case of the slightest suspicious finding during postoperative controls. It is always considered that postoperative changes may mask signs of malignancy. In this series, postoperative biopsy was performed in 6 cases (incisional biopsy in 2 cases, excisional biopsy in 4 cases). No cases were found to be malignant.

## Results

The cases included in the study are those with a minimum follow-up of 2 years and a maximum of 8 years. The average age of the patients was 42.9 years. The youngest patient was 19 years old and the oldest was 65 years old. Of the patients, 25 were under 40 years old. Preoperative USG evaluations showed that there was no pathology in both breasts of 43 patients. In 12 patients, benign pathologies such as fibrocystic changes, simple cysts, ductal ectasia, and lipomas were observed in 1 breast. Bilateral benign pathologies were detected in 9 patients. Biopsy was performed in 1 patient due to microcalcification in 1 breast, and reduction mammaplasty was performed when the pathology results were benign. Determining the location of benign pathologies in USG is also useful for the selection of the pedicle in the operation. For example, if the pathology is located in the inferior area, the superior, superolateral, or superomedial pedicle is selected and the pathology is tried to be included in the excision area.

**Graph 1.**
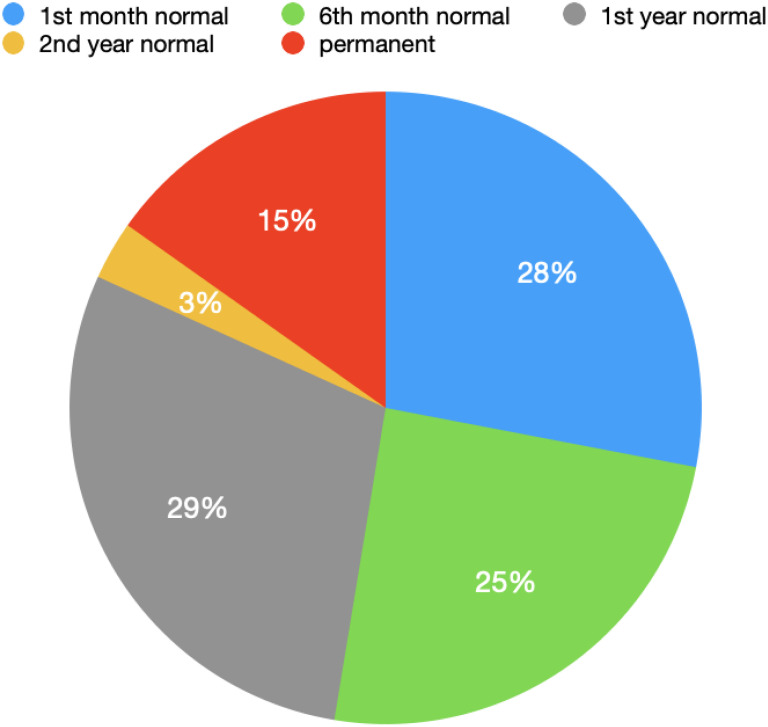
Graph showing the time it takes for postoperative changes to return to normal according to ultrasound results.

**Graph 2.**
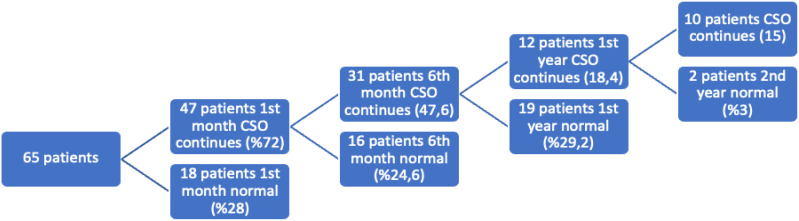
Graph showing the normalization of postoperative changes over time in 65 patients.

Changes secondary to the operation (CSO), such as increased skin and subcutaneous thickness, seroma, fat necrosis, and edema, were detected in 47 patients in the postoperative 1-month control with USG. Mastitis was detected and treated in 2 patients. The first-month USG result was normal in 18 patients. CSO returned to normal at the sixth month in 16 of 47 patients. At the first year, in 19 of 47 patients, and at the second year in 2 of 47 patients. Changes secondary to the operation was observed to become permanent in 10 patients. The results are shown in Graphs 1 and 2. Among the patients whose postoperative changes were permanent, in a 63-year-old patient, the area of fat necrosis in the right breast disappeared in the fourth year and was followed with a diameter of 18 mm in the left breast. Another patient, 47 years of age, was found to have fat necrosis in both breasts in the second year follow-up. Postoperative changes were observed as multiple simple cysts in 4 patients aged 36, 40, 48, and 51 years old, and MG was performed to follow up the condition of the cysts in terms of shape and size. Postoperative changes in 2 patients, aged 32 and 42 years, were followed up for 6 and 4 years, respectively, as fibrocystic changes. These patients were also included in MG during follow-up visits. The patients aged 20 and 32 years, who were found to have irregularity and calcification in the third and fourth year USG follow-up, were evaluated with MG and MRI, and biopsy was performed. Biopsy results revealed that the findings were benign in both patients.

In the first month follow-up, no CSO was observed in 28% of the patients, while it was observed in 72% of the patients. In the patients in whom these postoperative secondary changes persisted at the first month, CSO returned to normal at the sixth month in 24.6% of the patients, at the first year in 29.2% of the patients, and at the second year in 3% of the patients. Postoperative secondary changes were permanent in 15.3% of all patients. In total, CSO observed in USG returned to normal in 84.7% of the patients.

**Graph 3.**
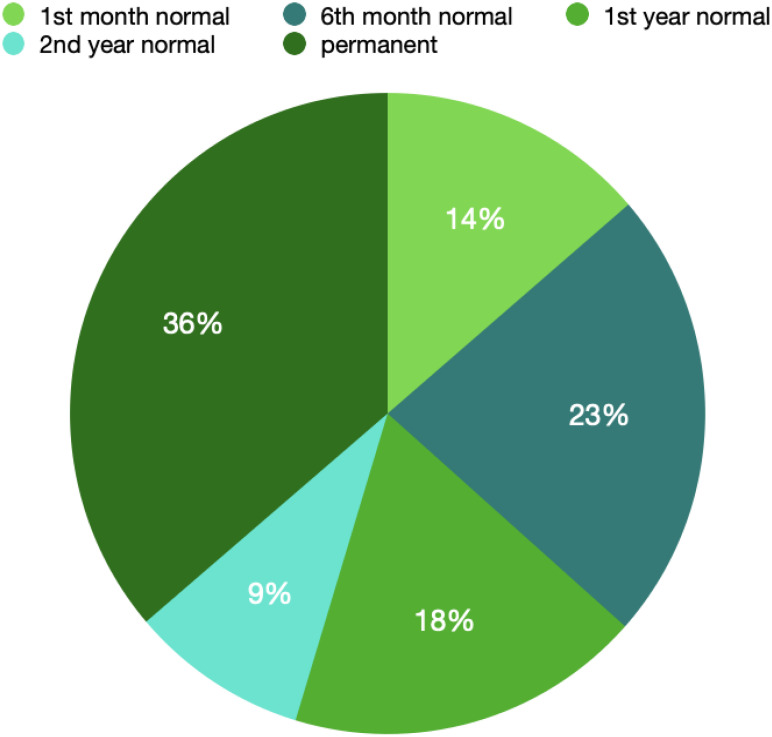
Graph showing the time to normalization of postoperative changes in patients with preoperative benign findings according to ltrasound results.

Benign lesions such as simple cysts, lipoma, fibrocystic lesions, and fibroadenoma were found in 22 patients in preoperative USG. The operation plan was made to remove these lesions. Of those, normal USG findings were observed in 3 patients at the first month, 5 patients at the sixth month, 4 patients at the first year, and 2 patients at the second year. It was permanent in 8 patients. The results are shown in Graph 3.

Mammography was added to the follow-up of all patients with preoperative benign lesions, and biopsy was performed after MRI in 2 patients. Biopsy results were benign.

All patients were carefully evaluated at their annual controls, even if postoperative changes have returned to normal. The patients with suspected malignancy in USG evaluations were evaluated with MG even if they were under 40 years of age, and MRI was requested when necessary. If the suspicion of malignancy cannot be ruled out, biopsy is recommended. In the follow-up of 4 patients whose secondary changes returned to normal, new lesions were observed to have developed. Biopsy was performed in these 4 patients because of malignty suspicion.

## Discussion

Reduction mammoplasty is one of the most frequently used precedures today in plastic surgery practice. Reduction mammoplasty is applied to normal breast tissue. The pathological examination of the removed breast tissues facilitates the diagnosis of occult BC. However, patients should be screened for BC that may occur in their continuing life. Radiological diagnostic tools should be used effectively so that the changes in the tissue after the surgery are not confused with the changes in BC. Recently, post-reduction mammaplasty changes have been described, especially in MG. The most common MG findings were parenchymal redistribution, elevation of the nipple, shift of the breast tissue to a lower position, calcifications, oil cysts, and fat necrosis.^9^

Ultrasound has long been used as a first-line assessment tool for early detection of BC. The USG findings of malignancy are well defined.^4^ Irregular shape, irregular margin, uncertain margin, microcalcification, and surrounding tissue distortion are often described as signs of malignancy.^7^^,^^8^ But there are no publications in the literature about specific USG findings after reduction mammaplasty. Ultrasound is frequently used in postoperative controls because it is economical and patient-friendly. Ultrasound with or without MG is the best tool for detecting early postoperative complications. Determining whether postoperative changes return to normal is useful in determining BC screenings of patients undergoing reduction mammaplasty.

The only real disadvantage of USG is that its success depends on the operator. As the number of RM operations increases, the number of evaluations increases, and specific USG findings are also determined. Thus, the USG assessments will become more successful and the results would be excellent.

## Figures and Tables

**Table 1.** Ultrasonographic Characteristics of Malignant and Benign Tumors

Feature	Malign tumor	Benign tumor
Shape	Irregular	Round, oval
Orientation	Usually vertical	Usually parallel
Margin	Uncertain	Evident, thin capsula
Margin contour	Irregular, angular, spiculate	Smooth, gentle lobulation
Echogenicity	Markedly hypoechoic	Hyperechoic, isoechoic
Geneity	Homogeneous	Heterogeneous
Posterior features	Shadowing	No changes, enhancement
Calcification	Microcalcification	No calcification
Surrounding tissue	Architectural distortion	Compression, no change
Retraction phenomenia	Present	Absent

## Data Availability

The data that support the findings of this study are available on request from the corresponding author.
